# Strophalloside Induces Apoptosis of SGC-7901 Cells through the Mitochondrion-Dependent Caspase-3 Pathway

**DOI:** 10.3390/molecules20045714

**Published:** 2015-03-31

**Authors:** Xue-Jiao Zhang, Wen-Li Mei, Guang-Hong Tan, Cai-Chun Wang, Song-Lin Zhou, Feng-Ru Huang, Bin Chen, Hao-Fu Dai, Feng-Ying Huang

**Affiliations:** 1 Institute of Tropical Bioscience and Biotechnology, Chinese Academy of Tropical Agricultural Sciences, Haikou 571199, China; E-Mails: 18889548680@163.com (X.-J.Z.); meiwenli@itbb.org.cn (W.-L.M.); 2 Hainan Provincial Key Laboratory of Tropical Medicine, Hainan Medical College, Haikou 571199, China; E-Mails: tanhoho@163.com (G.-H.T.); yctanyc@126.com (C.-C.W.); zhousonglin106@163.com (S.-L.Z.); frhuanghin@163.com (F.-R.H.); chenbinhin@163.com (B.C.); 3 College of Pharmacy and Chemistry, DaLi University, Dali 671099, China

**Keywords:** strophalloside, gastric carcinoma, mitochondrion-mediated pathway, apoptosis, cytochrome c, caspase

## Abstract

Cardenolides with special chemical structures have been considered as effective anti-cancer drugs in clinic trials. Strophalloside is a cardenolide we recently isolated from *Antiaris toxicaria* obtained from Hainan, China. The aim of this study was to investigate the possible anticancer effects induced by strophalloside and the underlying molecular mechanism. Gastric carcinoma SGC-7901 cells were treated with strophalloside at various concentrations for different times, and resulting cell viability was determined by the MTT assay, and the motility and invasion of tumor cells were assessed by the Transwell chamber assay. Apoptosis were measured by Annexin V-FITC/PI and Hoechst staining. The changes of mitochondrial transmembrane potential were examined by a JC-1 kit. The expressions of pro-apoptotic protein cytochrome c, caspase-3 and caspase-9 were detected by western blotting analysis. The results showed that strophalloside was capable of reducing cell viability, inhibiting cell growth, and suppressing cell migration and invasion in a time- and dose-dependent manner. Mitochondrial membrane potential declined and the concentration of cytochrome c increased in cytoplasm and caspase-3 and caspase-9 were cleaved into activated states, suggesting that cytochrome c was released from the mitochondrion to cytoplasm and finally activated the caspase-dependent apoptosis pathway. Our results indicate that strophalloside is a potential anticancer drug.

## 1. Introduction

The tropical rain forest is an important source of anti-cancer compounds. At present, several anti-cancer drugs were either isolated from the tropical rain forest or bear a close structural relationships to natural compounds [1,2,3].

**Figure 1:**
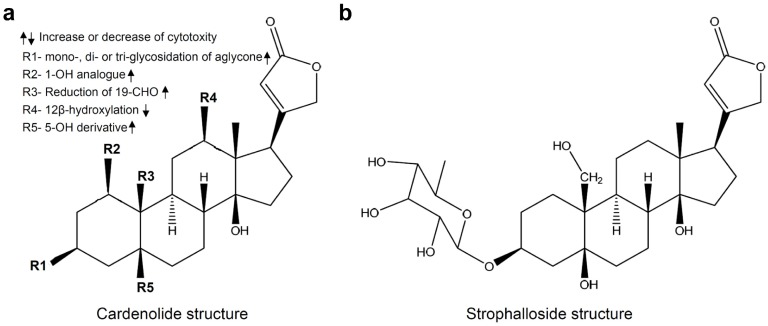
(**a**) The structure of cardenolides and a summary of structural features regarding the observed anti-cancer cytotoxicity; (**b**) the structure of strophalloside.

*Antiaris toxicaria* is widespread in the tropical rain forest of Southeast Asia. In China, it mainly grows in the warmer southern and eastern areas, such as Hainan, Guangdong, Guangxi, and Yunnan provinces. Its latex and seeds contain a complex mixture of cardenolides. For the development of tropical medicinal plant resources and under the guidance of activity screening, our laboratory has isolated a series of cardenolides from the latex, seeds, and stem of *Antiaris toxicaria* [4,5,6,7,8]. Strophalloside, one of these cardenolides isolated in our laboratory, has a special cardenolide structure that was first reported by our laboratory (Figure 1b). Traditionally, cardenolides are clinically used to treat congestive heart failure and arrhythmia [9,10,11]. However, recent research results have shown that certain cardenolidea extracted from natural sources have antitumor capabilities as they are capable of blocking tumor cell proliferation and inducing tumor cell apoptosis through regulation of several cell signaling pathways [12,13,14,15,16,17,18,19] and sodium pump inhibition [20].

Recent study results demonstrate that the antitumor cytotoxicity induced by cardenolides is highly related to their chemical structure. For example, the cardenolidea, in which pharmacophore R1 is a mono-, di- or tri-glycosidated aglycone, R2 is the 1-OH analogue, R3 is the reduced product of the 19-CHO moiety, and R5 is the 5-OH derivative, show higher antitumor cytotoxicity; but lower antitumor cytotoxicity is observed if the pharmacophore R4 is the 12β-hydroxylation product (Figure 1a) [21]. Strophalloside is a cardenolide we first isolated from the seeds of *Antiaris toxicaria*. Structure analysis shows that except for R4 the pharmacophores R1, R2, R3 and R5 in strophalloside are in complete agreement with these best structural characteristics, suggesting strophalloside is potentially a better anticancer drug. Thus, in this study, we tested its anticancer activities and its possible mechanism of action using the human gastric carcinoma cell line SGC-7901. We found that strophalloside could significantly inhibit SGC-7901 cell proliferation, migration and invasion, and induce SGC-7901 cell apoptosis at a low concentration. Subsequently, we further investigated the molecular mechanisms leading to apoptosis activation. The results demonstrated that strophalloside induced apoptosis in SGC-7901 cells through a mitochondrion-dependent mechanism.

## 2. Results and Discussion

### 2.1. Inhibition of SGC-7901 Cell Proliferation

As we know, uncontrolled proliferation, migration and invasion are the major malignant characteristics of cancer cells. In our present study, we first tested whether strophalloside inhibited SGC-7901 cell proliferation *in vitro* using the MTT assay. SGC-7901 cells were treated with strophalloside at various concentrations (0.12, 0.24, 0.47, 0.93 nM/mL, respectively) and cell viability was detected at 24 and 48 h after strophalloside treatment. As shown in Table 1, strophalloside significantly inhibited SGC-7901 cell proliferation in a dose- and time-dependent manner when compared with the control group (*p* < 0.001).

**Table 1:** Strophalloside inhibition of SGC-7901 cell proliferation.

Groups	Dose (nM/mL)	24 h		48 h	
		A Value	Inhibition (%)	A Value	Inhibition (%)
Control	0.0	0.92 ± 0.027	00.00 ± 00.00	0.975 ± 0.034 *	00.00 ± 00.00
Strophalloside	0.12	0.855 ± 0.029 *	7.06 ± 3.15 *	0.890 ± 0.056 *	8.71 ± 5.80 *
	0.24	0.782 ± 0.015 *	15.00 ± 1.60 *	0.747 ± 0.040 *	23.38 ± 4.23 *
	0.47	0.675 ± 0.009 *	26.63 ± 1.02 *	0.356 ± 0.012 *	62.56 ± 1.31 *
	0.93	0.465 ± 0.005 *	49.46 ± 0.50 *	0.104 ± 0.005 *	89.33 ± 0.57 *

Data of six independent experiments were expressed as mean ± SD. * *p* < 0.001 *vs.* control group.

### 2.2. Inhibition of SGC-7901 Cell Migration and Invasion

**Figure 2:**
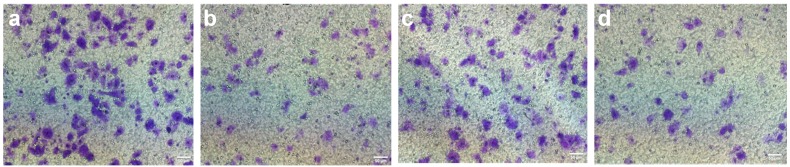
Representative images of cell migration and invasion in control and strophalloside-treated SGC-7901 cells detected by Transwell assays. (**a**) Migration in the control group; (**b**) Migration in the strophalloside-treated group (0.47 nM/mL); (**c**) Invasion in the control group; (**d**) Invasion in the strophalloside-treated group (0.47 nM/mL).

We used a Transwell chamber assay to test whether strophalloside inhibited SGC-7901 cell migration and invasion. Different concentrations of strophalloside (0.12, 0.24, 0.47, 0.93 nM/mL) were added into upper parts of the chambers, cultured in a 5% CO2 humidified incubator at 37 °C for 24 h and 48 h. The results of cell migration and invasion detected by Transwell assays are presented in Table 2 and Figure 2. Clearly, compared with the control group, the addition of strophalloside to the medium in the upper chamber resulted in significant suppression of SGC-7901 migration and invasion also in a dose-dependent manner at 0.12, 0.24, 0.47, and 0.93 nM/mL, respectively (*p* < 0.001).

**Table 2:** Strophalloside inhibition of SGC-7901 cell migration and invasion.

Groups	Dose (nM/mL)	Migration		Invasion	
		Cell Number	Inhibition (%)	Cell Number	Inhibition (%)
Control	0.0	153.1 ± 8.74	00.00 ± 00.00	140.8 ± 7.66	00.00 ± 00.00
Strophalloside	0.12	127.4 ± 6.52 *	16.79 ± 7.42 *	108.0 ± 5.47 *	23.30 ± 5.30 *
	0.24	76.7 ± 7.31 *	49.90 ± 6.31 *	64.2 ± 6.06 *	54.40 ± 4.29 *
	0.47	49.3 ± 6.47 *	67.80 ± 8.47 *	41.2 ± 5.97 *	70.74 ± 4.24 *
	0.93	30.7 ± 7.54 *	79.95 ± 5.75 *	22.4 ± 4.77 *	84.10 ± 3.39 *

Data of three independent experiments were expressed as mean ± SE. * *p* < 0.01 *vs.* control group.

### 2.3. Strophalloside Induced SGC-7901 Cell Death in Vitro

Apoptosis is a tightly regulated progress that is under the control of a series of gene regulation and cell-signaling pathways [22,23]. During apoptosis, cells undergo characteristic morphological and biochemical changes, accompanied by a specialized series of cellular events such as chromatin condensation, DNA fragmentation, cytoplasmic membrane blebbing, and cell shrinkage [24].

**Figure 3:**
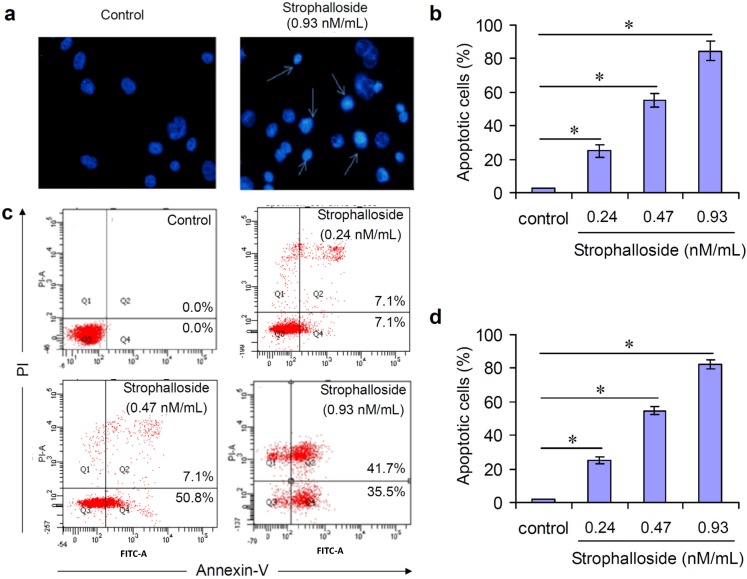
Induction of SGC-7901 cell death *in vitro*. (**a**) SGC-7901 cells were treated with 0.93 nM/mL) of strophalloside for 24 h and stained with Hoechst 33258. The apoptotic cells are indicated with arrows. Normal nuclear morphology is observed in untreated cells (control). In contrast, small, fragmented, and condensed nuclei with typical apoptotic morphology were observed in treated cells; (**b**) The percentage of apoptotic SGC-7901 cells was calculated in 10 hpf by Hoechst 33258 staining; (**c**) The characteristic images of flow cytometry analysis. SGC-7901 cells were treated with strophalloside (0.24, 0.47, 0.93 nM/mL) for 24 h and stained with FITC-conjugated annexin-V and propidium iodide (PI); (**d**) The percentage of apoptotic SGC-7901 cells was calculated in triplicate by flow cytometry analysis. * *p* < 0.001.

In this study, in order to know whether strophalloside treatment induced SGC-7901 cell death, Hoechst 33258 staining was used to observe the morphology of chromatin condensation and DNA fragmentation. Generally, apoptotic cells stained with Hoechst 33258 revealed typical apoptotic nuclei, which exhibited highly fluorescent condensed chromatin. Figure 3a,b show representative photomicrographs of SGC-7901 cells treated with or without strophalloside at a concentration of 0.93 nM/mL for 24 h. In control cultures, the nuclei of SGC-7901 cells appeared with regular contours and presented dark blue and uniform fluorescence, and cells with smaller nuclei and condensed chromatin were rarely seen (Figure 3a). In contrast, most of the nuclei of SGC-7901 cells treated with strophalloside appeared tight and hypercondensed (brightly stained), and the numbers of SGC-7901 cells with smaller nuclei and condensed chromatin increased significantly (Figure 3a). The percentage of the characteristic nuclei was counted at 10 high power fields (hpf), and the results showed that the percentage of characteristic apoptotic nuclei in the strophalloside-treated SGC-7901 cells was more than 80% (Figure 3b).

In the early stages of apoptosis, phosphatidylserine (PS) is translocated from the inner side of the plasma membrane to the outer layer. Annexin-V, a calcium dependent phospholipid-binding protein with a high affinity for PS, can therefore be used as a sensitive probe for the exposure of PS on the cell membrane and hence as a marker of apoptosis. In this study, flow cytometry analysis was used to detected phosphatidylserine exposure to the outside cell membranes. annexin-V^−^ and PI^−^ cells were designated as normal cells. In contrast, annexin-V^+^ and PI^−^ cells were designated as the apoptotic; and annnexin-V^+^ and PI^+^ cells were designated as the necrotic or late state of the apoptotic. SGC-7901 cells were treated with strophalloside at concentrations of 0.24, 0.47 and 0.93 nM/mL for 24 h. As shown in Figure 3c, flow cytometry analysis of the cell population showed distinct population sets. Total cell relative mortality (early apoptosis + late apoptotic cells and necrotic cells or G2 + G4) were counted as apoptotic cells and its percentage was calculated in triplicate. Compared with the control, significant apoptotic cells were seen in the strophalloside-treated SGC-7901 cells in a dose-independent manner (Figure 3d). Together with the results of the above Hoechst 33258 staining experiments, the results demonstrate that strophalloside can cause significant apoptosis of SGC-7901 cells *in vivo*.

### 2.4. Strophalloside Inhibited Tumor Growth and Induced SGC-7901 Cell Apoptosis in Vivo

**Figure 4:**
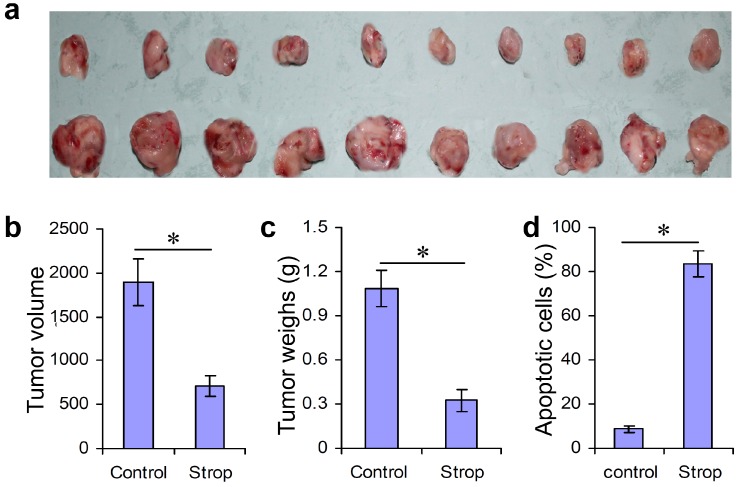

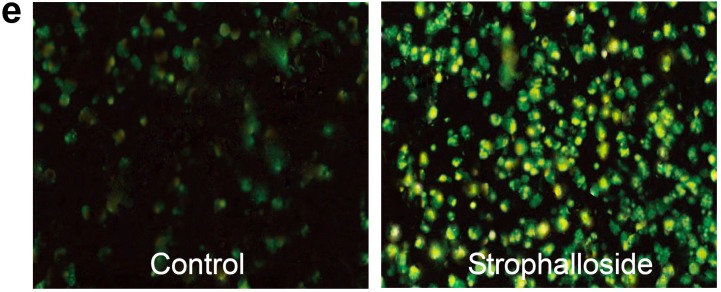
Inhibition of tumor growth and induction of SGC-7901 cell apoptosis *in vivo*. SGC-7901 tumor model was established in BALB/c nude mice and injected intravenously with strophalloside (Strop) or DMSO (Control) once every 4 days. On day 18 after tumor cell inoculation, the tumor masses were isolated. (**a**) The morphology of the tumor masses from the strophalloside-treated mice (upper) and control mice (lower); (**b**) The tumor volumes; (**c**) The tumor weights; (**d**) The percentage of the apoptotic cells calculated from (**e**); (e) The tumor masses from the strophalloside-treated and the control were sectioned and stained with *In Situ* Cell Death Detection Kit (fluorescein, Roche). * *p* < 0.001.

Although we know that strophalloside treatment of SGC-7901 cells induced significant cell apoptosis *in vitro* in the above experiments, the most convincing evidence that could facilitate clinical translational usage of strophalloside as an anticancer drug is the *in vivo* determination of activity in a mouse tumor model. In this study, we further established a SGC-7901 tumor model in BALB/c nude mice. These mice were then injected intravenously with strophalloside (93.3 nM/kg per mouse, dissolved in 100 μL DMSO) or control solution (50 μL DMSO) every four days. At day 18 after SGC-7901 cells were inoculated, the tumor masses were directly removed and we recorded the tumor volume and weight. The results demonstrated that the tumor masses from the strophalloside-treated mice were smaller than those from the control mice (Figure 4a). In addition, significantly decreased tumor volume (Figure 4b) and weight (Figure 4c) were found in the mice treated with strophalloside, but not in the mice treated with the control DMSO. Moreover, considering apoptosis is the major mechanism of action of most chemical anti-cancer drugs, we used the tumor masses to prepare frozen sections stained with the *In Situ* Cell Death Detection Kit (fluorescein, Roche, Basle, Switzerland). In this method, the apoptotic tumor cells in the tumor tissues were stained with green fluorescence that indicated the nuclei of the apoptotic cells were positively stained. Figure 4e shows the characteristic staining of control and strophalloside-treated mice. More than 80% of the tumor cells in the tumor tissues from the strophalloside-treated mice showed apoptosis at day 18 after strophalloside treatment four times (Figure 4d).

### 2.5. Strophalloside Activated the Mitochondrion-Mediated Apoptosis Pathway

At present, two distinct but convergent pathways can initiate apoptotic responses: the death receptors and mitochondrial pathways [25,26,27]. The death-receptor pathway is activated when death ligands—TNF-Related Apoptosis-Inducing Ligand (TRAIL), Fas Ligand (FasL), and Tumor Necrosis Factor α (TNF-α)—bind to cell surface death receptors members of the TNF Receptor (TNFR) family. Binding of these receptors causes the downstream activation of caspase-8, which directly activates other members of the caspase family and triggers the execution of apoptosis [28,29,30]. However, the mitochondrial pathway of cell apoptosis is regulated through caspase-9 [31,32,33]. Initiators of caspase-9 ultimately lead to increased mitochondrial permeability, thereby facilitating the release of cytochrome c (Cyt c) from the inter-mitochondrial membrane space into the cytosol [34,35,36]. Cyt c ultimately results in the activation of caspase-9 through the apoptosome, triggering the activation of caspase-3 and resulting in cell death. Thus, both activated caspase-8 (death receptor pathway) and caspase-9 (mitochondrial pathway) in turn mobilize caspase-3 (the key executioner caspase) can finally cause cell apoptosis [28,37].

**Figure 5:**
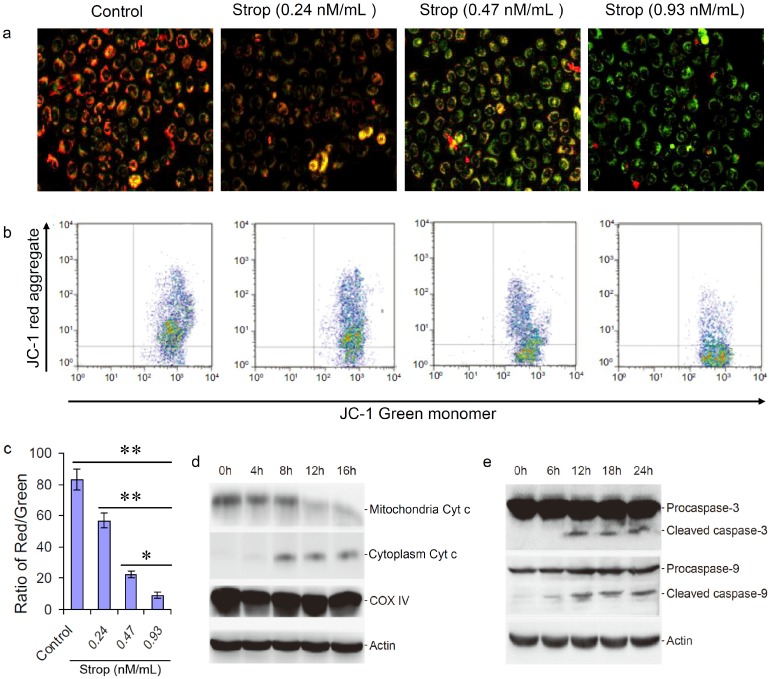
Changes of the mitochondrial transmembrane potential and caspase activation by mitochondrial Cyt c. (**a**) The mitochondrial transmembrane potential was tested using JC-1 fluorescent staining of SGC-7901 cells treated with different concentrations of strophalloside (Strop); (**b**) The characteristic images of flow cytometry analysis of the red (aggregate) and the green (monomer); (**c**) The ratio of JC-1 aggregates and monomers were calculated in triplicates by flow cytometry analysis; (**d**) Strophalloside treatment induced mitochondrial Cyt c released into cytoplasm; (**e**) Mitochondrion-pathway-related caspase-3 and caspase-9 were activated from procaspases into cleaved states, which were detected by western blotting analysis. The results of (d, e) showed that Cyt c was released from chondriosome and activation of capase-3 and capase-9. * *p* < 0.05 and ** *p* < 0.001.

The loss of mitochondrial membrane potential is a hallmark of apoptosis. It is an early event coinciding with caspase activation. In addition, the release of Cyt c from mitochrondria in response to proapoptotic signals has been suggested as an initiating event in the apoptotic process [36]. In order to evaluate whether the strophalloside-induced SGC-7901-cell apoptosis is related to a mitochrondrial pathway, we used JC-1 to detect the changes of mitochondrial transmembrane potential. In apoptotic and necrotic cells, JC-1 exists in monomeric form and stains the cytosol green. However, in normal cells, JC-1 accumulates as aggregates in the mitochondria which appear red. In this study, SGC-7901 cells were treated with strophalloside (0.24, 0.47 and 0.93 nM/mL) for 12 h and stained with JC-1. Compared with the control cells, the strophalloside-treated SGC-7901 cells showed significant green fluorescence (Figure 5a) and the green fluorescence intensity ratio increased dramatically and in a dose-dependent manner (Figure 5b). The results indicated that strophalloside caused a significant decrease in mitochondrial membrane potential in the SGC-7901 cells.

The current view is that the decrease of mitochondrial membrane potential is one of the earliest events in mitochondrion-mediated apoptosis, which causes the release of Cyt c from the mitochondria and initiates the mitochondrion-mediated apoptosis signaling pathway. In this study, we used western blot analysis to detect the caspase-9 and caspase-3 activities, which are the two important downstream molecules of the mitochondrion-mediated apoptosis signaling pathway. Our results showed that the released Cyt c from mitochondrion activated caspase-9 and caspase-3. Western blot analysis revealed that the level of Cyt c increased in the cytoplasm and decreased in the mitochondria of the SGC-7901 cells treated with strophalloside (0.93 nM/mL) for different times compared to the control (Figure 5d), suggesting strophalloside caused the release of Cyt c into the cytoplasma from the mitochondria. In addition, a significant increase in caspase-3 and caspase-9 activities (procaspase cleaved into active state) were observed at later time points in the cells treated with strophalloside (Figure 5e). Taken together, these data demonstrated that strophalloside-induced SGC-7901 cell apoptosis is related to damage to the mitochondria and promotion of the Cyt c release from mitochondria that activates the mitochondrion-mediated apoptosis pathway.

## 3. Experimental Section

### 3.1. Isolation and Purification of Strophalloside

Seeds of *Antiaris toxicaria* (Pers.) Lesch were collected in Lingshui County of Hainan Province, China, in May 2009, and the plant was identified by Professor Zhu-NianWang. A voucher specimen (No. AN200905) has been deposited in the Institute of Tropical Bioscience and Biotechnology, Chinese Academy of Tropical Agricultural Sciences. The dried and crushed seeds (25.9 kg) of *Antiaris toxicaria* were extracted with 95% EtOH (90 L) three times at room temperature. The combined extract (98 L) was evaporated *in vacuo* to yield the EtOH extract (590 g), which was suspended in H_2_O and then partitioned successively with petroleum ether (3 × 3 L), EtOAc (3 × 3 L), and *n*-BuOH (3 × 2.5 L). The *n*-BuOH fraction (169 g), which showed potent cytotoxity, was subjected to macroporous resin D-101 column chromatography eluting with H_2_O and MeOH to yield a MeOH fraction (79 g). The MeOH fraction was chromatographed over silica gel CC under reduced pressure and eluted with a gradient solvent system of CHCl_3_–MeOH (50:1, 30:1, 15:1, 10:1, 7:1, 4:1, 2:1, 1:1, 0:1, each 1.5 L) to give nine corresponding fractions (Fr.1–Fr.9). Fr.5 (13.4 g) was further separated by silica gel column chromatography, from which strophalloside was obtained. Its structure was confirmed in our previous report [11].

### 3.2. Cell Culture

Human gastric cancer cell line SGC-7901 was purchased from the Cell Bank of Type Culture Collection of Chinese Academy of Sciences, Shanghai Institute of Cell Biology (Shanghai, China). Cells were cultured in RPMI 1640 medium supplemented with 10% FBS, 100 IU/mL penicillin and 100 mg/mL streptomycin at 37 °C in a humidified atmosphere with 5% CO_2_. Cells at the logarithmic phase were used for experiments.

### 3.3. Measurement of Cell Viability

An MTT assay was used to examine the growth and viability of SGC-7901 cells. For the MTT assay, SGC-7901 cells in logarithmic growth were trypsinized and harvested and then the cells were seeded onto a 96-well plate. After 24 h, fresh RPMI 1640 medium containing different concentrations of strophalloside (0.12, 0.24, 0.47, 0.93 nM/mL) was added at 100 μL per well, respectively, and six replicate wells were used for each of the concentrations. After incubation for different time intervals, 10 μL of MTT solution (5 mg/mL) was added to each well and the cells were further incubated at 37 °C for 4 h. The supernatant was then removed and 100 μL DMSO was added into each well. Absorbance (A value) at a wavelength of 490 nm was measured with a Bio-TekEXL808 microplate reader (Bio-Tek, Winooski, VT, USA).

### 3.4. Invasion and Migration Assay

Invasion assays were performed in a 24-well Transwell chamber (Corning, Lowell, MA, USA). Briefly, each Transwell chamber was coated with 10 μg Matrigel, and 1 × 10^5^ cells were seeded on pre-coated filters in 200 μL of serum-free medium containing different concentrations of strophalloside (0.12, 0.24, 0.47, 0.93 nM/mL) in triplicate. The lower parts of the chambers were filled with 700 μL of RPMI 1640 medium containing 20% FBS. After incubation in a 5% CO_2_ humidified incubator at 37 °C for 24 h, the cells on the upper surface were gently removed with a cotton swab, and the filters were fixed with 4% paraformaldehyde for 20 min and stained with 0.1% crystal violet for 20 min. The number of cells on the lower surface of the filters was quantified under a microscope. The same procedures were followed for the migration assay except the Transwell chambers were not coated with Matrigel.

### 3.5. Nuclear Morphology Analysis

Apoptotic cells were stained with Hoechst 33258 dye and assessed by fluorescence microscopy. Briefly, after SGC-7901 cells were treated with strophalloside to the experimental time points, the cells were harvested, fixed with absolute ethanol, and stained with Hoechst 33258 for 20 min at 37 °C. The cells were then visualized using fluorescence microscopy (80i, Nikon, Tokyo, Japan) with UV excitation at 300–500 nm. Cells containing condensed and/or fragmented nuclei were considered to be apoptotic.

### 3.6. Flow Cytometric Analysis of Apoptotic Cell

The extent of apoptosis was measured through an AnexinV-FITC apoptosis detection kit (Beyotime Institute of Biotechnology, Beijing, China) as described in the manufacturer’s instruction. After cells were exposed to drugs for 24 h, then they were collected and washed with PBS twice, gently resuspended in annexin-V binding buffer and incubated with annexinV-FITC/PI in dark for 15 min and analyzed by flow cytometry using cell quest software (BD Biosciences, Franklin Lakes, NJ, USA). The fractions of cell population in different quadrants were analyzed using quadrant statistics. Cells in the lower left quadrant represented survivals; lower right quadrant represented apoptosis and in the upper right quadrant represented necrosis or post-apoptotic necrosis.

### 3.7. Establishment of SGC-7901 Tumor Model and Strophalloside Treatment

The animal protocols involved in this study were approved by the Animal Care and Use Committee of Hainan Medical College. Twenty BALB/c nude mice at 6 to 8 weeks of age were subcutaneously injected with 2 × 10^6^ live SGC-7901 cells in the right flank to establish tumor models. Thereafter, mice were randomly divided into control and strophalloside-treated group of 10 mice in each group. The strophalloside dose and treatment protocol was referred to our previous study [38]. Strophalloside-treated mice were injected intravenously by tail vein with strophalloside (93.3 nM/kg, dissolved in 100 μL dimethylsulfoxide (DMSO)) once every four days; the control mice were injected with 100 μL DMSO once every three days. Eighteen days after tumor cells were inoculated, the tumor masses were surgically removed and observed, and the tumor volume and weight were recorded.

### 3.8. Detection of Tumor Cell Apoptosis in Vivo

An immunohistochemistory method was used to study SGC-7901 cell apoptosis in tumor tissues *in situ*. Frozen tumor masses isolated from the strophalloside-treated or control mice were prepared in 3–5 μm sections. Thereafter, these sections were fixed with 1% paraformaldehyde in PBS, and apoptotic cells were detected with Terminal-deoxynucleoitidyl Transferase Mediated Nick End Labeling (TUNEL) staining according to the manufacturer’s instructions (*In Situ* Cell Death Detection Kit; Fluorescein; Roche). Apoptosis was quantified by determining the percentage of positively stained cells for all of the nuclei in 20 randomly chosen fields/section at 200× magnification. Slides of the apoptosis studies were quantified in a blind fashion by two independent reviewers two different times.

### 3.9. Mitochondrial Membrane Potentials Assay

JC-1 probe was employed to measure mitochondrial depolarization in SGC-7901 cell. Briefly, Cells cultured in six-well plates after indicated treatments were incubated with an equal volume of JC-1 staining solution (5 μg/mL) at 37 °C for 20 min and rinsed twice with PBS. Mitochondrial membrane potentials were monitored by determining the relative amounts of dual emissions from mitochondrial JC-1 monomers or aggregates using a Nikon (80i, Tokyo, Japan) fluorescence microscope under argon-ion 488 nm laser excitation. Mitochondrial depolarization is indicated by an increase in the green/red fluorescence intensity ratio. For fluorescence-activated cell sorting (FACS) study, the treated cells were washed twice with PBS (pH 7.4) and collected by centrifugation, followed by incubating with JC-1 working solution for 20 min at 37 °C in 5% CO_2_ humidity. Then, the cells were resuspended in 500 μL 1× incubation buffer. Thereafter, flow cytometry was performed to detect the red/green fluorescence with a FACS Canto II (BD Biosciences) and the data were analyzed with the BD FCSDiva Software (V6).

### 3.10. Preparation of Cytosolic and Mitochondrial Extracts

Cytoplasma and mitochondria proteins were collected using a cell mitochondrial kit from Beyotime in accordance with the manufacturer’s instructions. Briefly, The harvested pellets incubated in cell lysis buffer (250 mM of sucrose; 1 mM of DTT; 10 mM of KCl; 1 mM of EDTA; 1 mM of ethyleneglycolbis(aminoethyl ether)-tetraacetic acid (EGTA); 1.5 mM of MgCl_2_; phenylmethylsulfonyl fluoride; 20 mM of HEPES, pH 7.4) at 4 °C and homogenized with a glass homogenizer. The cell lysate was centrifuged at 800× *g* for 10 min to remove any unbroken cells, and the supernatant was further centrifuged at 15,000× *g* for 10 min at 4 °C. The resulting supernatant contained the cytoplasmic fraction and the pellet contained the mitochondrial fraction. The mitochondrial pellet was further resuspended in a mitochondrial lysis buffer at 4 °C, and both the resuspended mitochondrial pellet solution and the supernatant were further centrifuged at 24,000× *g* at 4 °C to remove the nuclei. The protein concentration of the cytoplasma and mitochondria was measured by the Bradford method using BSA as a standard.

### 3.11. Western Blot Analysis

Western blot analysis was performed as we previously reported [39]. Equal amounts of protein (40 μg/sample) was separated electrophoretically by 10%–12% SDS-PAGE and blotted onto PVDF membranes. The blots were probed with the primary antibodies and incubated with a horseradish peroxidase-conjugated anti-IgG in blocking buffer for 1 h. After washing, the blots were developed with ECL (Santa Cruz Biotechnology, Santa Cruz, CA, USA) and exposed to X-ray film (Eastman-Kodak, Rochester, NY, USA). The polyclonal antibodies specific to β-acctin (1:1000), COX IV (1:1000), caspase-3 (1:250), cytochrome c (1:200), and caspase-9 (1:1,000) were purchased from Santa Cruz Biotechnology. The polyclonal antibody specific to β-actin (1:500 dilutions) was purchased from Beyotime.

### 3.12. Statistical Analysis

The significance of differences between experimental groups was analyzed using ANOVA and/or the unpaired Student’s *t*-test. Data were expressed as mean ± SD, *p-*values < 0.05 were considered significant.

## 4. Conclusions

The present work described the apoptosis-inducing effects of strophalloside in a gastric carcinoma cell line (SGC-7901). The *in vitro* results showed that strophalloside decreased cell viability, inhibited cell growth, and suppressed cell migration and invasion in a time- and dose-dependent manner. In an *in vivo* SGC-7901 tumor model, treatment with strophalloside induced significant inhibition of tumor growth and induction of significant tumor cell apoptosis. Mitochondrial membrane potential, one of the earliest events in mitochondrion-mediated apoptosis, remarkably declined in the strophalloside-treated SGC-7901 cells. In addition, the concentration of cytochrome c decreased in mitochondria, but increased in cytoplasm. Moreover, caspase-3 and caspase-9 were cleaved into active states after strophalloside treatment. These data suggested that cytochrome c was released from the mitochondria to the cytoplasm and finally activated the caspase-dependent apoptosis pathway. Our results indicate that strophalloside was a potential anticancer drug.
